# On the pinning force in high density MgB_2_ samples

**DOI:** 10.1038/s41598-021-85209-2

**Published:** 2021-03-15

**Authors:** V. Sandu, A. M. Ionescu, G. Aldica, M. A. Grigoroscuta, M. Burdusel, P. Badica

**Affiliations:** National Institute of Materials Physics, Street Atomistilor 405A, 077125 Magurele, Romania

**Keywords:** Superconducting properties and materials, Composites, Ceramics

## Abstract

An analysis of the field dependence of the pinning force in different, high density sintered samples of MgB_2_ is presented. The samples were chosen to be representative for pure MgB_2_, MgB_2_ with additives, and partially oriented massive samples. In some cases, the curves of pinning force versus magnetic field of the selected samples present peculiar profiles and application of the typical scaling procedures fails. Based on the percolation model, we show that most features of the field dependence of the critical force that generate dissipation comply with the Dew-Hughes scaling law predictions within the grain boundary pinning mechanism if a connecting factor related to the superconducting connection of the grains is used. The field dependence of the connecting function, which is dependent on the superconducting anisotropy, is the main factor that controls the boundary between dissipative and non-dissipative current transport in high magnetic field. Experimental data indicate that the connecting function is also dependent on the particular properties (e.g., the presence of slightly non-stoichiometric phases, defects, homogeneity, and others) of each sample and it has the form of a single or double peaked function in all investigated samples.

## Introduction

Magnesium diboride, MgB_2_, is one of the most exciting superconductors discovered in the last two decades due to a series of advantages that makes it attractive for applications. It has also a very interesting physics that brings it in the spotlight among other high temperature superconductors. One of the most important properties is the capacity to transport a high super current in an applied magnetic field. The analysis of this process showed that grain boundaries act as the main pinning structure though other mechanisms could not be neglected. The analysis of the field *B* and temperature *T* dependence of the pinning force *F*_p_ = *J*_c_ × *B* can provide important information on the mechanisms involved in the pinning process. For metallic, low temperature superconductors, Dew-Hughes^1^ showed that the field dependence of *F*_p_ obeys the general law:

1 $${F}_{p}= K{h}^{p}{\left(1-h\right)}^{q}$$

where *h* is the reduced magnetic field *h* = *H*/*H*_c2_ with *H*_c2_ the upper critical field and *K* a constant. The exponents *p* and *q* depend on the pinning mechanism and on the dimension of the pinning manifold. Table 1 shows the value of the exponents according to the Dew-Hughes model^1^. Moreover, the plots of reduced pinning force *f*_p_ = *F*_p_/*F*_p,max_ vs. *h* at different temperatures, with *F*_p,max_ being the maximum value of *F*_p_(*h*), would peak at ${h}_{p}=\frac{p}{p+q}$ and collapse on the same curve. However, if this scaling seems to work for some low temperature superconductors, its validity for the new classes of superconductors is unclear and the attempts to fit *f*_p_(*h*) data using the exponents given in the Table 1 were not always successful^2^. Many puzzling results on this topic are reported for superconducting MgB_2_, single crystals, ceramics, and tapes^3–10^. An analysis of the limitations of this model was presented in the Ref.^11^. Several authors tried to circumvent this drawback using a series of the type ${f}_{p}=\sum_{i}{A}_{i}{h}^{{p}_{i}}{\left(1-h\right)}^{{q}_{i}}$ with *p*_i_ and *q*_i_ from the Table 1. Besides the fact that the physics beneath such a direct summation of different mechanisms is questionable, the exponents *p*_i_ and *q*_i_ proved to be different from those predicted in Table 1^12,13^. Ihara and Matsushita^14^ proposed a Pythagorean summation for the associated critical current density when several types of pinning contribute. In that case *f*_p_ is depicted as ${f}_{p}=B{\left(\sum_{i}{J}_{ci}^{2}\right)}^{1/2}$.

**Table 1:** Exponents of the pinning force.

Type of pinning		*p*	*q*
Point pins	Core pinning	1	2
	δ*k* pinning	2	1
Surface pins	Core pinning	1/2	2
	δ*k* pinning	3/2	1
Volume pins	Core pinning	0	2
	δ*k* pinning	1	1

Considering that the pinning force is related to the critical current density, the effort was driven to find hints for the field dependence of *J*_c_ using different combinations of *H*, *J*_c_ and different derivatives of *J*_c_ leading to a linear dependence. However, these combinations seemed to work only in a limited field range, thus, introducing two or three crossover fields. If different field-related regimes can be valid in superconducting cuprates, where the interplay between weak pinning, short coherence length, and long penetration depths generate different regimes of the collective pinning^15^, it would raise difficulties regarding their interpretation in the case of MgB_2_ with a much longer coherence length and stronger pinning.

In this paper, we investigate the field dependence of the pinning force in MgB_2_ high density samples obtained by spark plasma sintering (SPS). We selected a series of samples whose field dependence of the pinning force strongly depends on the additives and on the procedures applied to the green samples. It is an attempt to find the common features of the pinning and of the reasoning behind the dependence between the parameters *p*, *q* and the temperature.

## Methods

Five bulk samples of high density magnesium diboride, pure or containing small amounts of additives, were prepared by spark plasma sintering (SPS) technique. The mass density of the samples is higher than 95% of the ideal MgB_2_. The details of raw materials, additives, preparation conditions, as well as the structure, microstructure and physical properties of the samples are presented in the references attached to each sample. The samples selected for analysis are: (*i*) pure MgB_2_^16^; (*ii*) (MgB_2_)_0.99_(Te_0.25_(HoO_1.5_)_0.75_)_0.01_^17^; (*iii*) (MgB_2_)_0.99_(B_4_C)_0.01_^18^; (*iv*) weakly oriented MgB_2_ (orientation degree ~ 21%)^19^; (*v*) highly oriented MgB_2_ (orientation degree ~ 40.5%)^20^. The partial *c*-axis orientation was induced in the green compacts of the samples (*iv*) and (*v*) by field assisted slip casting (FASC) under a high magnetic field of 12 T. The subsequent SPS procedure enhanced the orientation.

Samples were cut from the center of the sintered disc with a diameter of 2 cm and a thickness of 0.4 cm. The size of the randomly oriented samples (*i*)–(*iii*) was 1.5 × 1.5 × 0.5 mm^3^, while the partially-oriented samples were 1 mm^3^ cubes. The magnetization loops at different temperatures (5–35 K) of the as-prepared samples were measured by using a MPMS-7 T magnetometer (Quantum Design). The field dependence of *J*_c_ was determined with the Bean model. For all the samples, the macroscopic irreversibility field was used as the scaling field instead of *H*_c2_. The irreversibility field was obtained from the field dependence of the critical current density with the criterion *J*_c_(*H*_irr_) = 100 A cm^−2^.

## Results

**Figure 1:**
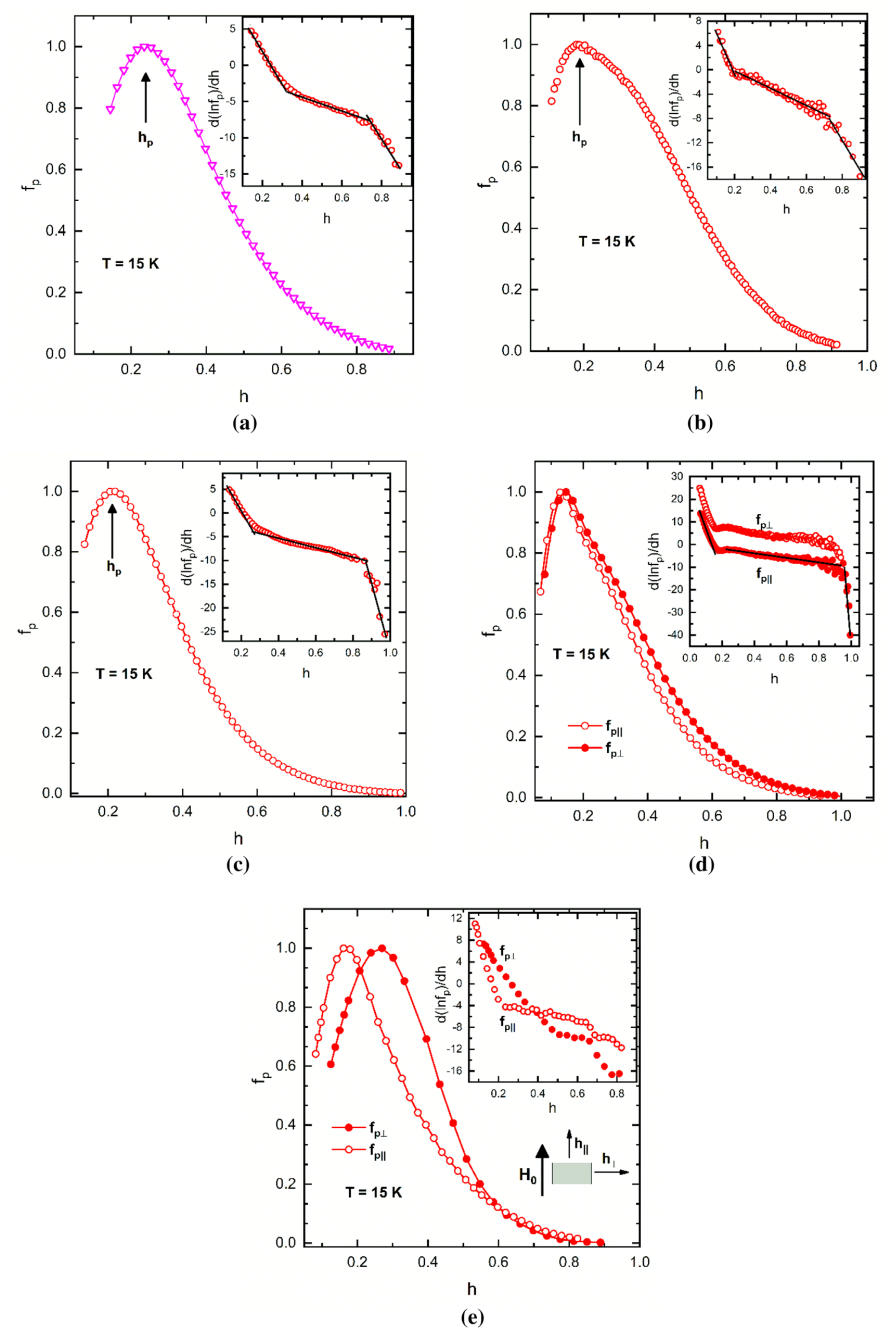
Dependence of the reduced pinning force *f*_p_ and their logarithmic derivatives $\frac{d\left({\text{ln}}{f}_{p}\right)}{dh}$ (Insets) on the reduced field *h* at 15 K: (**a**) pure MgB_2_ (*i*); (**b**)- (MgB_2_)_0.99_(Te_0.25_(HoO_1.5_)_0.75_)_0.01_ (*ii*); (**c**) (MgB_2_)_0.99_(B_4_C)_0.01_ (*iii*); (**d**) weakly oriented MgB_2_ (*iv*); and (**e**) highly oriented MgB_2_ samples (*v*). The geometry of measurements is presented in the second inset to image (**e**) measurements (*H*_0_ the applied field during slip casting procedure and *h*_||_, *h*_⊥_ are the reduced measuring fields). In the inset to (**d**), the plot of *f*_p⊥_ is shifted upwards with 8 units. In all plots, *h*_p_ stands for the peak point. Lines in the Insets are guide for the eye.

Figure 1 shows the dependence of the reduced pinning force *f*_p_ on the reduced field *h* for all five samples at the same temperature *T* = 15 K. Following the suggestion of Ref.^21^, the plots of $\frac{dln\left({f}_{p}\right)}{dh}$ vs. *h* are shown in the insets. They were interpreted as consisting of three linear parts which implies two crossover fields. It is worthy to note that as-obtained linearity would suggest a Gauss-like *h*-dependence of *f*_p_.

A closer examination of these plots shows that the position of the peak of *f*_p_(*h*) is dependent on the samples’ features for a given temperature in a large *h*-range. For example, at *T* = 15 K, the value of *h*_p_ spans from *h*_p_ = 0.13 for the weakly oriented sample (*iv*) measured in perpendicular geometry (Fig. 1d) to *h*_p_ = 0.26 for the highly oriented one (*v*) measured in parallel geometry (Fig. 1e). The only samples showing a peak at a *h*-value close to the theoretical one of *h*_p_ = 0.20 for the grain boundary pinning are (*ii*) and (*iii*) added with Te/Ho_2_O_3_ and B_4_C, respectively. It is remarkable that in the sample (*ii*) there are no substitutions in the crystal structure of MgB_2_, while in the sample (*iii*) carbon supplied from B_4_C substitutes for boron. Another observation of interest is that in the samples doped with tellurium and rare earth oxide, (MgB_2_)_0.99_(Te_*x*_(HoO_1.5_)_*y*_)_0.01_^17^, *h*_p_ shifts to lower values with increasing ratio *y*/*x*. For example, at T = 5 K, *h*_p_ = 0.15 for the sample with the composition (MgB_2_)_0.99_(Te_0.25_(HoO_1.5_)_0.75_)_0.01_ but *h*_p_ = 0.19 for (MgB_2_)_0.99_(Te_0.31_(HoO_1.5_)_0.69_)_0.01_. Such values of *h*_p_ smaller than the theoretically predicted ones were previously reported by other groups^22,23^.

Other features noticeable in some samples are a shoulder and/or several inflection points (Fig. 1b,d). These details are easily visible on the graph of the derivative d(*f*_p)_)/d*h*. Shoulders and inflections were also reported by other authors^24^.

A third peculiarity is the anisotropy of *f*_p_ which is displayed by the partially-oriented samples (*iv*) and (*v*). The crystallographic texture leads to a noticeable difference between the reduced pinning forces *f*_p||_ and *f*_p⊥_. These reduced pinning forces were obtained with the measuring field applied along and perpendicular to the *c*-axis of MgB_2_. The anisotropy is small in the case of weakly oriented sample (*iv*) (Fig. 1d) with the peak fields *h*_p||_= 0.13 and *h*_p⊥_ = 0.145. In the case of highly oriented sample (*v*), the anisotropy is stronger and the difference between the peak fields is significant with *h*_p||_= 0.26 and *h*_p⊥_ = 0.17 (Fig. 1e) (for measurement geometry see the inset 2 of Fig. 1e).

Finally, we mention the shift of *h*_p_ to higher values with increasing temperature. It was interpreted as a crossover to pinning on other manifolds. Though, the dominant pinning elements in MgB_2_ are the grain boundaries and obviously they do not disappear with the increasing temperature.

**Figure 2:**
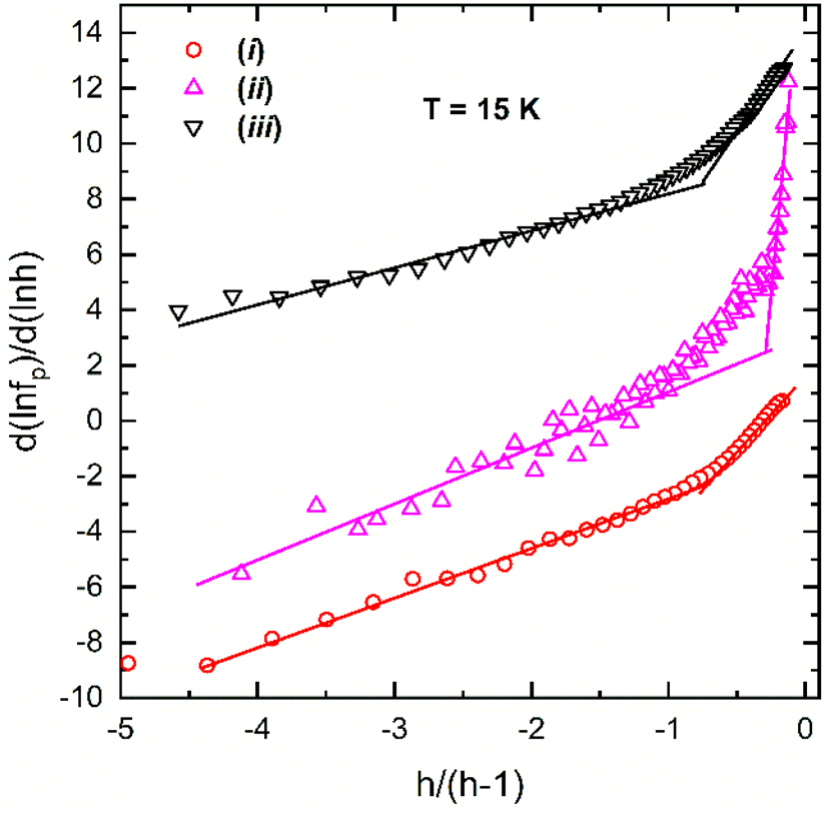
Dependence of the logarithmic derivative of the reduced pinning force *f*_p_ on the rescaled field *h*/(*h*-1) measured at 15 K for sintered MgB_2_ samples: (*i*) MgB_2_; (*ii*) (MgB_2_)_0.99_(Te_0.25_(HoO_1.5_)_0.75_)_0.01_; (*iii*) (MgB_2_)_0.99_(B_4_C)_0.01_. The data for samples (*ii*) and (*iii*) are shifted upwards with 6 and 12 units, respectively. Lines are guides for the eye.

A first attempt to investigate the field dependence of *f*_p_(*h*) for our samples was to start from the Dew-Hughes assumption and to use the reduced form of Eq. (1). Parameters *p* and *q* were determined. Specifically, we plotted the logarithmic derivative $\frac{d\left({\text{ln}}{f}_{p}\right)}{d\left({\text{ln}}h\right)}$ vs. *x* = *h*/(*h* − 1) which, if the assumption is correct, the plot would be linear providing the exponents *p* and *q* representing the slope and intercept, respectively. Examples of the indicated plot are shown in Fig. 2 for the samples (*i*)–(*iii*) measured at 15 K. The curves suggest the existence of at least two field regimes with a crossover at a certain field *h*_c_ where the slope changes. However, the as-determined parameters *p* and *q* do not correspond to any known pinning regime. Thus, for *h* < *h*_c_, *q* takes abnormally high values in the range 4 ≤ *q* ≤ 44, whereas for *h* > *h*_c_, *p* is negative. For the samples plotted in Fig. 2, we obtained the following values: *p* = 1.67, *q* = 5.35 for *h* < *h*_c_ and *p* = − 1.59, *q* = 1.64 for *h* > *h*_c_ in pure MgB_2_ (sample (*i*)); *p* = 10, *q* = 44 for *h* < *h*_c_ and *p* = -4, *q* = 1.3 for *h* > *h*_c_ in (MgB_2_)_0.99_(Te_0.25_(HoO_1.5_)_0.75_)_0.01_ (sample (*ii*)); and *p* = 1.33, *q* = 5.1 for *h* < *h*_c_ and *p* = − 3.22, *q* = 1.12 for *h* > *h*_c_ in (MgB_2_)_0.99_(B_4_C)_0.01_ (sample (*iii*)).

These plots, as well as other combinations of field which were made in an attempt to obtain the linear representation suggest a complex field dependences of the pinning force. We remind that the pinning force is in fact the result of the field dependence of the critical current density *J*_c_. Consequently, different, more or less evasive mechanisms were invoked to explain the field dependence. There were attempts to apply collective pinning models although their validity was proved to be correct in the case of the cuprate superconductors, but it is questionable for MgB_2_. Actually, bulk superconductors, and especially MgB_2_, have a very complex structure acquired during processing depending on technology specifics and on the nature of the ingredients.

A MgB_2_ bulk sample is a collection of superconducting grains which also include non-superconducting phases like MgO and higher magnesium borides, and voids. Moreover, the superconducting grains themselves might have defects. Among them we mention vacancies (mainly of Mg), substitutions (e.g. of C for B) and inclusions, all of them being responsible for the local critical parameters. In a magnetic field, the structural anisotropy plays also an important role because the superconducting properties of each grain depend on the orientation relative to the applied field. In this landscape, the supercurrent paths are very complex and vary with temperature and field. To approach this problem, a *percolation model* was developed by Eisterer et al.^25–27^. According to this model, the critical current density *J*_c_(*H*) is given by^27^:

2 $${J}_{c}\left(H\right)=\underset{0}{\overset{{J}_{c,M}}{\int }}{\left[\frac{{p}_{\sigma }\left(J\right)-{p}_{c}^{*}}{1-{p}_{c}^{*}}\right]}^{t}{\left(\frac{{p}_{s}-{p}_{c}}{1-{p}_{c}}\right)}^{t}dJ$$

where *J*_*c,M*_(*H*) is the maximum *J*_*c*_ for the material, *p*_*σ*_(*J*) is the fraction of the dissipation free material at a given *J* among the superconducting grains, *p*_*s*_ is the fraction of MgB_2_, *p*_*c*_ is the percolation threshold, ${p}_{c}^{*}={p}_{c}/{p}_{s}$ and *t* = 1.76. Thus, the unavoidable presence of insulating phases and voids increases the effective percolation threshold to ${p}_{c}^{*}$ which can be expressed as ${p}_{\sigma }({J}_{c,M}) ={p}_{c}^{*}$. The fraction of dissipation free material *p*_*σ*_ (*J*) decreases with increasing *J* due to the variation of the local irreversibility field from grain to grain. However, there is a minimal current density *J*_c,m_ below which *p*_*σ*_ = 1 so that Eq. (2) can be written as

3 $${J}_{c}\left(H\right)={\left(\frac{{p}_{s}-{p}_{c}}{1-{p}_{c}}\right)}^{t}{J}_{c,m}+\underset{{J}_{c,m}}{\overset{{J}_{c,M}}{\int }}{\left[\frac{{p}_{\sigma }\left(J\right)-{p}_{c}^{*}}{1-{p}_{c}^{*}}\right]}^{t}{\left(\frac{{p}_{s}-{p}_{c}}{1-{p}_{c}}\right)}^{t}dJ$$

Further, we consider a polycrystalline bulk sample made of grains with both similar anisotropy* γ* and superconducting properties. Consequently, the irreversibility field of each grain depends on the orientation *θ* relative to the applied field. For the angular dependence of the irreversibility field, Matsushita et al.^28^ proposed a dependence similar to the upper critical field, i.e., ${H}_{\text{irr}}\left(\theta \right)=\frac{{H}_{irr}\left(\pi /2\right)}{\sqrt{{\gamma }^{2}{\text{cos}}^{2}\theta +{\text{sin}}^{2}\theta }}$, whereas a more complex dependence is obtained if the zero-resistivity field is considered ${H}_{\text{irr}}\left(\theta \right)=\frac{{H}_{c2}\left(\pi /2\right)}{\sqrt{\left({\gamma }^{2}{\text{cos}}^{2}\theta +{\text{sin}}^{2}\theta \right)\left[\left({\gamma }^{2}-1\right){p}_{c}^{2}+1\right]}}$^27^. However, the former expression is more suitable for a single grain while the latter seems more appropriate for the percolative transport. In both cases, if the pinning on grain boundary is considered, *J*_c,m_ and *J*_c,M_ are given by:

4a $${J}_{c,m}\propto \left\{\begin{array}{c}{\left(1-H/{H}_{\text{irr}}(0)\right)}^{2}{\left(H/{H}_{\text{irr}}\left(0\right)\right)}^{-1/2} , H\le {H}_{irr}\left(0\right)\\ 0 , H>{H}_{irr}\left(0\right)\end{array}\right.$$

4b $${J}_{c,M}\propto {\left(1-H/{H}_{\text{irr}}\right)}^{2}{\left(H/{H}_{\text{irr}}\right)}^{-1/2}$$

In Eq. (4b), *H*_irr_ is field that breaks the last supercurrent carrying path, i.e., ${p}_{\sigma }({H}_{irr}) ={p}_{c}^{*}$. Consequently, *H*_irr_(0) < *H*_irr_ < *H*_irr_(π/2) even though disconnected grains displaying irreversibility still survive in the field range *H*_irr_ < *H* ≤ *H*_irr_(π/2). Considering Eq. (3), the critical force *F*_p_ = μ_0_*HJ*_c_, which defines the dissipation onset and which will be further called the *pinning force*, gets the form:

5 $${F}_{p}\left(H\right)={\mu }_{o}H\left\{A\left(1,{p}_{c},{p}_{s}\right){J}_{c,m}+\underset{{J}_{c,m}}{\overset{{J}_{c,M}}{\int }}A\left({p}_{\sigma }\left(J\right), {p}_{c},{p}_{s}\right)dJ\right\}$$

where *A*(*p*_σ_(*J*), *p*_c_, *p*_s_) is the integrand of Eq. (2), with ${p}_{\sigma }({J}_{c,M}) ={p}_{c}^{*}$. The *F*_p_ depends on the real pinning through the local critical current, but, macroscopically, the non-dissipative transport is controlled by percolation. Because *A*(*p*_σ_(*J*), *p*_c_, *p*_s_) is a monotonous decreasing function of *p*_σ_, hence, of *J*, applying the mean value theorem of integration^29^ one obtains:

6 $${F}_{p}\left(H\right)={\mu }_{0}H{J}_{c,M}A\left(\stackrel{\sim }{p}, {p}_{c}, {p}_{s}\right)\left\{1+\left[\frac{A\left(1,{p}_{c},{p}_{s}\right)}{A\left(\stackrel{\sim }{p}, {p}_{c}, {p}_{s}\right)}-1\right]\frac{{J}_{c,m}}{{J}_{c,M}}\right\}$$

where $\stackrel{\sim }{p}$ is a value between *p*_c_ and *p*_σ,max_(*H*), the maximal value of *p*_σ_ at a given field *H*, i.e., the fraction of grains for which *H* < *H*_irr_. The *J*_c,M_, is related to the macroscopic irreversibility field. The *p*_max_(*H*) might be extracted from the angular distribution of the grains *G*(*θ, ϕ*), which gives ${p}_{\sigma }\left(\theta \right)={\int }_{\theta }^{\pi /2}{\int }_{0}^{2\pi }G\left(\theta {^{\prime}},\varphi {^{\prime}}\right){\text{sin}}\theta {^{\prime}}d\theta {^{\prime}}d\varphi {^{\prime}}$, and the angle dependence of *H*_irr_ if the right form of both *G*(*θ, ϕ*) and of *H*_irr_(*θ*) is known. However, an analytical form for *p*_σ_(*θ*) can be obtained only for a constant angular distribution^26^.

The integrand in Eq. (5), hence, $A\left(\stackrel{\sim }{p}, {p}_{c}, {p}_{s}\right)$ is a decreasing function of *H* no matter the angle distribution, number of phases or percolation thresholds. In fact, Eq. (5) is helpful to determine the high field (decreasing) part of *F*_p_(*H*). The low field dependence raises more problems than it could suggest the simple form which appears as the second term in the brackets of Eq. (6). Dew Hughes^1^ proposed a local decrease of the shear modulus at grain boundaries that would lead to an alignment of the vortices along the boundaries. Possible plastic deformations, if appear, might lead to dissipation only if percolative channels develop^30^. However, as the elastic moduli of the vortex lattice are also dependent on the orientation of vortices relative to the crystalline axes and anisotropy, the saturation of the synchronization is reached at different fields for different orientation and depends on the grain distribution and the presence of different superconducting phases. In the absence of a model that should describe such a complex process we propose to use a field dependent factor, similar to the efficiency factor proposed by Dew Hughes^1^, that can be experimentally determined. In addition, the distribution of the irreversibility fields is required in real samples because the irreversibility is dependent on grain size ^31^.

A general form for the reduced pinning force *f*_p_ = *F*_p_/*F*_p,max_ in terms of reduced field *h* = *H*/*H*_irr_ can be obtained from the Eqs. (5) and (6) interpolated to the low field factor and averaged on grain size. In addition to the form proposed by Dew Hughes, it contains a field dependent coupling factor *g*(*h*,*T*) in polycrystalline samples that arise from the anisotropy of the samples and can be determined from the experimental data:

7 $${f}_{p}\left(h, T\right)= {h}^{1/2}{\left(1-h\right)}^{2}g\left(h, T\right)$$

This equation has the advantage to preserve the same exponents *p* and *q*, hence, the pinning nature in the almost entire temperature range where *H*_irr_ (*T*) > 0. The function *g*(*h*,*T*) can account for the shift of the peak, the increase of the width, and for other peculiarities of *f*_p_: these effects emerge as the consequence of the percolative nature of the supercurrent transport.

The attempts to fit *f*_p_(*h*) experimental curves with Eq. (7) showed that *g*(*h*,*T*) is either a single or a double peaked function which depends on the sample composition and fabrication technique. These functions have the characteristics of a distribution function either Gaussian or lognormal. The reason for such a dependence is not clear and further investigations are required. Below, we present the data on *f*_p_(*h*,*T*) (symbols) and their fits with Eq. (7) (continuous lines) above in the temperature range 5–30 K for all samples discussed above.

**Figure 3:**
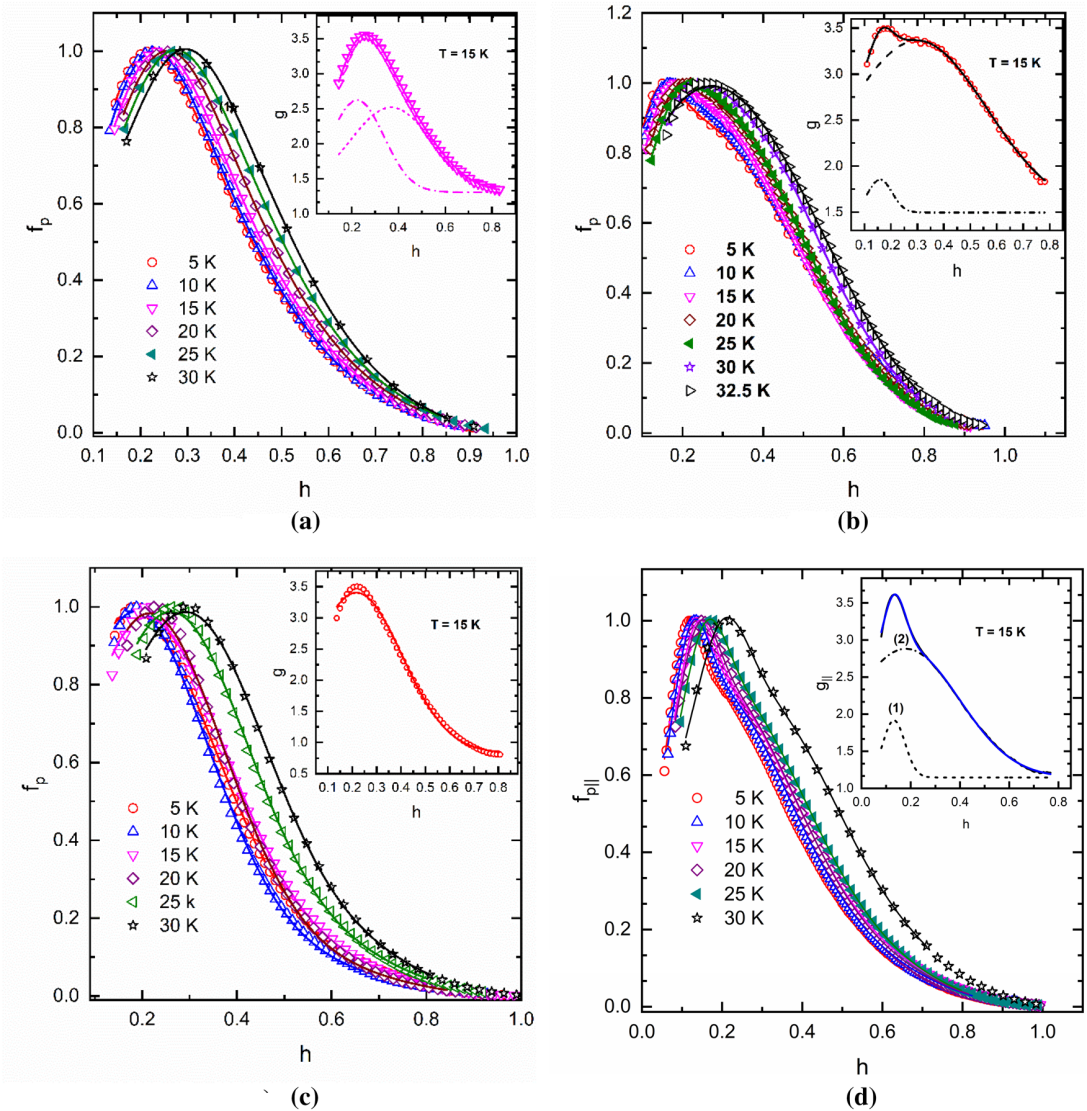
The dependence of the reduced pinning force *f*_p_ on the reduced magnetic field and the fits with Eq. (2) (continuous lines) in the temperature range 5–30 K: (**a**) pure MgB_2_ (sample (*i*)); (**b**) (MgB_2_)_0.99_(TeO_0.25_(HoO_1.5_)_0.75_)_0.01_ (sample (*ii*)); (**c**) (MgB_2_)_0.99_(B_4_C)_0.01_ (sample (*iii*)); (**d**) partially textured MgB_2_ (sample (*iv*)). Insets show the *h*-dependence of the function *g* at *T* = 15 K. Continuous line is the fit with the distribution function. Dash and dash dotted lines are the decomposition of the double peaked functions.

Figure 3a shows data for the sample (*i*) made of pure MgB_2_. In this case, *g*(*h*) is a double peaked Gaussian function, $g\left(h\right)={g}_{0}+\frac{{A}_{1}}{{\sigma }_{1}\sqrt{2\pi }}{\text{exp}}\left[-\frac{1}{2}{\left(\frac{h-{h}_{p1}}{{\sigma }_{1}}\right)}^{2}\right]+\frac{{A}_{2}}{{\sigma }_{2}\sqrt{2\pi }}{\text{exp}}\left[-\frac{1}{2}{\left(\frac{h-{h}_{p2}}{{\sigma }_{2}}\right)}^{2}\right]$ with slightly different amplitudes, *A*_1_ and *A*_2_, and standard deviation, *σ*_1_ and *σ*_2_, for each peak (See the inset to Fig. 3a for *T* = 15 K). This type of a double peaked Gaussian was also found for the more complex compositions corresponding to sample (*ii*) (Fig. 3b) and to the weakly oriented sample (*iv*) (Fig. 3d). The two samples have a different weight of each peak (see the insets to both figures). In the case of the sample (*iii*) doped with B_4_C, *g*(*h*) is a single peaked Gaussian (inset to Fig. 3c).

**Figure 4:**
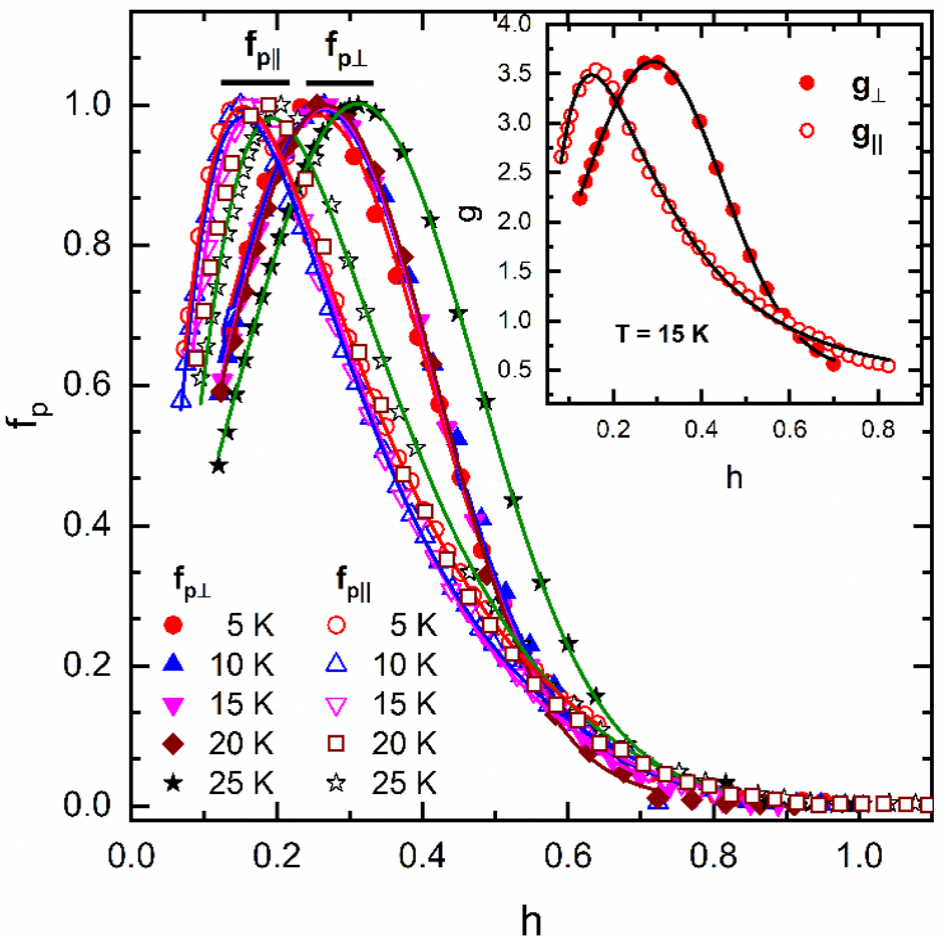
The dependence of the reduced pinning force *f*_p_ on the reduced magnetic for the partially oriented MgB_2_ sample (*v*) as measured in the parallel, *f*_p||,_ and perpendicular, *f*_p⊥_, geometry in the temperature range 5–25 K. Continuous lines are the fits with Eq. (2). Insets present *h*-dependence of the functions *g*_||_ and *g*_⊥_ at *T* = 15 K. Continuous line is the fit with a Gaussian for *g*_||_ and a lognormal function for *g*_⊥_.

More interesting is the case of the strongly oriented sample (*v*) (Fig. 4) for which *g*_||_(*h*) is a Gaussian and g_⊥_(*h*) is a lognormal function $g\left(h\right)={g}_{0}+\frac{A}{\sigma h\sqrt{2\pi }}{\text{exp}}\left\{-{\left[\frac{{\text{ln}}\left(h/{h}_{p}\right)}{\sigma \sqrt{2}}\right]}^{2}\right\}$ (see the inset to Fig. 4). In the case of the (MgB_2_)_0.99_(B_4_C)_0.01_ sample (*iii*) (Fig. 3c) and of the strongly textured sample (*v*) (Fig. 4), the use of only a single peaked distribution function can be roughly understood as a result of the grains orientation. The need of a double peaked *g*(*h*) in the case of the samples (*i*), (*ii*), and (*iv*) might indicate the presence of two types of MgB_2_ grains with slightly different intrinsic properties (anisotropy, local irreversibility field). For example, such phases can result from gradual spatial distribution of carbon (intended or accidental doping) due to its diffusion from the grain boundaries to the core of the MgB_2_ grains. The fitting parameters for all samples as determined at 15 K are given in Table 2.

**Table 2:** Fit data of the connection functions *g*(*h*) of all samples at *T* = 15 K.

Sample		*g*(*h*)	*g*_0_	*A*_1_	*A*_2_	*σ*_1_	*σ*_2_
(*i*)		Double Gaussian	1.31 ± 0.01	0.38 ± 0.21	0.53 ± 0.24	0.11 ± 0.02	0.18 ± 0.02
(*ii*)		Double Gaussian	1.5 ± 0.04	0.041 ± 0.006	1.23 ± 0.07	0.045 ± 0.002	0.262 ± 0.008
(*iii*)		Gaussian	0.83 ± 6 × 10^–15^	1.21 ± 1 × 10^–14^	–	0.19 ± 1 × 10^–15^	–
(*iv*)	*g*_||_	Double Gaussian	1.15 ± 0.01	0.086 ± 0.006	0.95 ± 0.03	0.045 ± 0.001	0.218 ± 0.004
	*g*_⊥_	Double Gaussian	0.74 ± 0.01	0.084 ± 0.007	1.18 ± 0.04	0.041 ± 0.001	0.222 ± 0.004
(*v*)	*g*_||_	Gaussian	0.50 ± 0.02	1.23 ± 0.02	–	0.157 ± 0.001	–
	*g*_⊥_	LogNormal	0.38 ± 0.04	1.15 ± 0.02	–	0.296 ± 0.015	–

**Figure 5:**
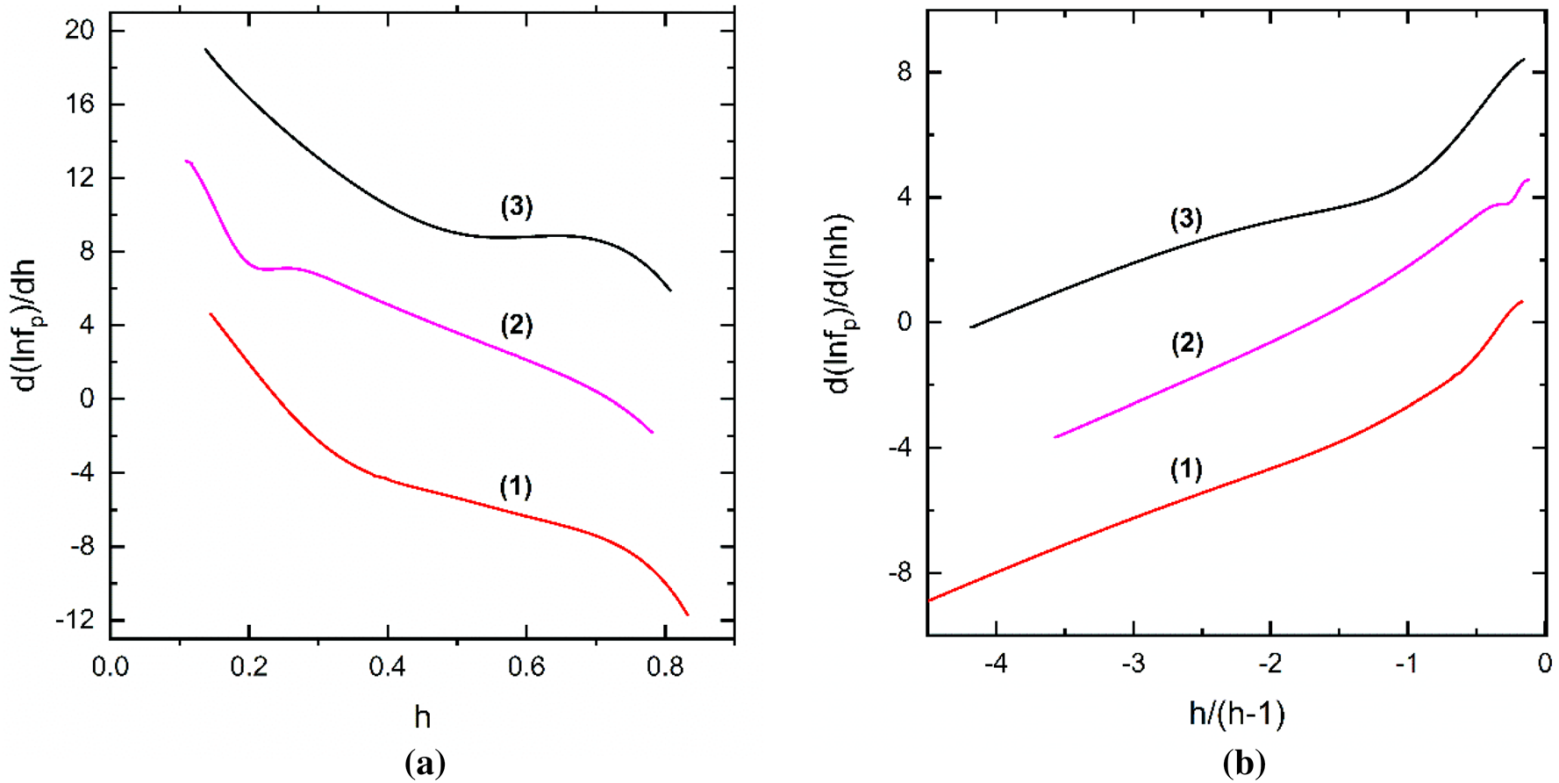
The dependence of the derivative of the fit functions of *f*_p_ with Eq. (6) at *T* = 15 K. The curves are for: (1) (*i*) pure MgB_2_; (2) (*ii*) (MgB_2_)_0.99_(Te_0.25_(HoO_1.5_)_0.75_)_0.01_; (3) (*iii*) (MgB_2_)_0.99_(B_4_C)_0.01_. Panel (**a**) shows *h*-dependence of logarithmic derivative of *f*_p_, $\frac{d\left({\text{ln}}{f}_{p}\right)}{dh}$. The plots (2) and (3) are shifted upward with 8 and 16 units, respectively. Panel (**b**) presents dependence of $\frac{d\left({\text{ln}}{f}_{p}\right)}{d\left({\text{ln}}h\right)}$ vs. *x* = *h*/(*h* − 1). The plots (2) and (3) are shifted upward with 4 and 8 units, respectively.

Equation (7) explains in a consistent way the peculiarities of the *h*-dependence of the derivative $\frac{d\left({\text{ln}}{f}_{p}\right)}{dh}$ and the shape of $\frac{d\left({\text{ln}}{f}_{p}\right)}{d\left({\text{ln}}h\right)}$ vs. *x* = *h*/(*h* − 1) curves as were shown in the Insets to Fig. 1 and in Fig. 2, respectively. Thus, Fig. 5a and b show the plots of $\frac{d\left({\text{ln}}{f}_{p}\right)}{d\left({\text{ln}}h\right)}$ vs. *x* = *h*/(*h* − 1) for the non-oriented samples as obtained with Eq. (7).

We mention that our procedure encounters difficulties around *h* ~ 1, i.e., for applied fields in the vicinity of *H*_irr_ where the data are scattered and the result is uncertain. Additional phenomena also must be taken into account close to *H*_irr_ where creep is strongly emphasized and proliferation of non-superconducting areas occurs.

In literature, the use of a distribution function was proposed to represent the voltage-current characteristics of high temperature superconductors. Namely, in Refs.^32^ and^33,34^ the distribution functions to describe the local critical current density were of a Gaussian or Weibull type, respectively.

## Conclusion

We have shown that the reduced pinning force *f*_p_ dependence on the reduced field *h* can be described in the case of polycrystalline bulk samples by the model of pinning on grain boundaries. A connecting function is associated and it arises from the peculiar structure of each sample.

At high fields, this function is the result of the percolation processes that are characteristic for the samples with intrinsic anisotropy and distribution of the orientation of the grains. It also mirrors the local properties of the grains as they result from their size, stress, doping, and inclusions. At lower fields, the manifestation of polycrystallinity was included in a field dependent factor similar to the efficiency factor used to illustrate the pinning in isotropic materials.

These properties are typical for sintered MgB_2_ samples, but the model might be suitable and applied also to other superconductors. The proposed model preserves the framework of the grain boundary pinning. It also removes the putative crossovers inferred from the behavior of different combinations of the field, current, and/or of their derivatives as well as the need for the models consisting of the summation of different pinning mechanisms.
